# Successful treatment with fecal microbiota transplantation in patients with multiple organ dysfunction syndrome and diarrhea following severe sepsis

**DOI:** 10.1186/s13054-016-1491-2

**Published:** 2016-10-18

**Authors:** Yanling Wei, Jun Yang, Jun Wang, Yang Yang, Juan Huang, Hao Gong, Hongli Cui, Dongfeng Chen

**Affiliations:** Department of Gastroenterology, Institute of Surgery Research, Daping Hospital, Third Military Medical University, Chongqing, 400042 China

**Keywords:** MODS, Fecal microbiota transplantation, Therapeutic efficacy, Diarrhea

## Abstract

**Background:**

The dysbiosis of intestinal microbiota plays an important role in the development of gut-derived infections, making it a potential therapeutic target against multiple organ dysfunction syndrome (MODS) after sepsis. However, the effectiveness of fecal microbiota transplantation (FMT) in treating this disease has been rarely investigated.

**Methods:**

Two male patients, a 65-year-old and an 84-year-old, were initially diagnosed with cerebellar hemorrhage and cerebral infarction, respectively, after admission. During the course of hospitalization, both patients developed MODS, septic shock, and severe watery diarrhea. Demographic and clinical data were collected. Intestinal dysbiosis was confirmed by 16S rDNA-based molecular analysis of microbiota composition in fecal samples from the two patients. The two patients each received a single nasogastric infusion of sterile-filtered, pathogen-free feces from a healthy donor. Fecal samples were collected every two days post infusion to monitor changes in microbiota composition in response to treatment.

**Results:**

Following FMT, MODS and severe diarrhea were alleviated in both patients. Their stool output and body temperature markedly declined and normalized. Significant modification of microbiota composition, characterized by a profound increase of commensals in the Firmicutes phylum and depletion of opportunistic organisms in the Proteobacteria phylum, was observed in both patients. Furthermore, we identified a reconstituted bacterial community enriched in Firmicutes and depleted of Proteobacteria that was associated with a decrease in the patients’ fecal output and in the levels of plasma inflammation markers.

**Conclusions:**

The outcome of treating two patients with FMT indicates that restoration of the intestinal microbiota barrier can alleviate the infection and modulate the immune response. These findings warrant further investigation of FMT as a putative new therapy for treating microbiota-related diseases such as MODS.

## Background

The normal function of the gastrointestinal tract is important for immune defense against pathogens in the human body. It is now well-acknowledged that gut microbiota may serve as a physical barrier that maintains mucosal integrity by preventing penetration of the epithelial barrier by pathogens and by modulating immunological activity [1]. Disruption of the gut microbiota barrier contributes to the development of many gastrointestinal diseases along with multiple extraintestinal diseases [2]. Fecal microbiota transplantation (FMT) has been proposed as a treatment for restoring the gut microbiota barrier [3, 4]. Recently, it has been shown that FMT can cure recurrent *Clostridium difficile* infection (CDI), which results from persistent disruption of commensal gut microbiota, by reestablishing the intestinal microbiota balance [5]. However, the nature of this restoration and whether a transition to an ecologically stable intestinal microbial population takes place during FMT treatment remains to be elucidated.

Multiple organ dysfunction syndrome (MODS), which in most cases occurs secondary to severe sepsis or septic shock, trauma, neoplastic diseases, and other causes of systemic inflammatory response syndrome (SIRS), refers to the presence of impaired function in multiple organs so that homeostasis cannot be maintained without medical intervention [6]. The pathophysiology of MODS is complex, multifactorial, and poorly understood. Emerging evidence suggests that translocation of microbes or components of microbes from the gastrointestinal tract and immune system dysregulation might be involved [7]. Thus, FMT might play a therapeutic role in the management of MODS following severe sepsis. The efficacy of FMT in treating recurrent CDI further encouraged us to investigate the value of this therapeutic approach in patients with MODS after sepsis. In this article, we describe two cases of patients who developed MODS and severe diarrhea following severe sepsis and report their outcomes following treatment with FMT. We also investigated the changes in composition and abundance of intestinal bacteria in these patients with MODS in response to FMT and characterized the relationship between these FMT-induced alterations in gut microbiota and immunological marker profiles.

### Case presentation

Case 1: a 65-year-old male patient with sudden loss of consciousness and an initial diagnosis of encephalorrhagia was transferred to our hospital from another medical facility. Cerebral computed tomography (CT) confirmed the diagnosis of cerebellar hemorrhage. This patient was then admitted to the Department of Neurosurgery, and the cerebellar hematoma was surgically removed via the posterior middle approach. After surgery, the patient was in a persistent light coma, but defecation was normal. At 6 days post surgery, the patient was passing yellow pasty stool once a day, but this frequency later increased to 3–4 times a day.

On postoperative day 8, the patient developed pulmonary infection and progressed to acute respiratory distress syndrome (ARDS), then suffered type I respiratory failure. This patient went into a coma after the operation and had intermittent irritability and much airway mucus; therefore, it was considered that the pulmonary infection was more severe. Thus, in order to aspirate the sputum and maintain airway patency, we carried out a tracheotomy. The patient was admitted to the intensive care unit (ICU) with a diagnosis of septic shock and MODS. The acute physiology and chronic health evaluation (APACHE) II score was 23, and the white blood cell (WBC) count increased to 26.22 × 10^9^/L with 84.2 % neutrophils. His blood cultures now yielded *Acinetobacter baumannii*. The patient had a persistently high fever, with a peak temperature of 39.6 °C, despite administration of antibacterial and antifungal therapy. Meanwhile, the sputum culture showed mycotic infection, so oral itraconazole was added. However, the patient’s condition continued to worsen.

On the 20th day of hospitalization, the patient developed septic shock. Antibiotics were changed to imipenem/cilastatin and vancomycin, while itraconazole was continued. The patient then had progressive diarrhea on average 6–12 times a day, with a total volume of 1000–2200 mL per day, with stool of a pasty jam consistency. We carried out comprehensive bacteriological investigations, including stool bacterial culture and fungal culture, and tested for *Clostridium difficile* toxin A and B. All results were negative. The fecal bacteria population was analyzed, and the results indicated severely perturbed intestinal microflora. Based on the above findings, this patient was rendered a good candidate for treatment with FMT. Therefore, FMT was performed once, and the anti-infection therapy was stopped.

Case 2: an 84-year-old male patient with right-sided weakness and fever was admitted. CT confirmed the presence of cerebral infarction near the left lateral ventricle and pulmonary infection. The patient was then transferred to ICU due to infection-induced respiratory failure. His blood cultures now yielded *Burkholderia cepacia*. Antibiotics were changed from imipenem/cilastatin to cefoperazone/sulbactam. On the fourth day of hospitalization, fluconazol was added due to the discovery of pseudohyphae in the sputum. The pneumonia improved, but the patient suffered from fever and diarrhea after the use of multiple antibiotics. The anti-infection therapy was ineffective. In addition, metronidazole, probiotics, and loperamide were useless in treating the diarrhea. Consequently, the patient’s fever persisted, and he progressed to MODS. His APACHE II score was 20. Continuous hemofiltration was given for aggravated renal failure on the seventh day of hospitalization. The patient had diarrhea 4–8 times a day, with a total stool volume of 1000–2000 mL per day. We carried out comprehensive bacteriological investigations for this patient too, including stool bacterial culture and fungal culture, and tested for *C. difficile* toxin A and B. All results were negative. As was the case for the first patient, analysis of the fecal bacteria indicated severe microflora imbalance and thus, a likelihood of successful treatment with FMT. Based on the experience of treating the first patient, the same FMT regimen was administered to this patient.

## Methods

### Analysis of fecal microbiota

To confirm our suspicion of intestinal dysbiosis, we used 16S rRNA gene-based molecular techniques to characterize the fecal bacterial composition in our two patients according to the following procedure [8]. DNA was extracted from fecal samples, stored at −80 °C, and later purified by standard methods. DNA density and quality were evaluated using the NanoDrop spectrophotometer (ThermoFisher Scientific-NanoDrop Products, Wilmington, DE, USA), Qubit fluorometer (ThermoFisher Scientific-Life Technologies, Grand Island, NY, USA), and agarose gel electrophoresis. Extracted DNA was diluted to 2 ng/μL and stored at −20 °C until sequencing of 16S gene amplicons.

Universal primers (5′-GTACTCCTACGGGAGGCAGCA-3′ and 5′-GTGGACTACHVGGGTWTCTAAT-3′) with 8 nt index barcodes were used to amplify the V3V4 hypervariable regions of 16S rRNA genes for subsequent sequencing using the MiSeq sequencer (Illumina, San Diego, CA, USA) [9, 10]. The PCR mixture (25 μL) contained 1 × PCR buffer, 1.5 mM MgCl_2_, 0.4 μM of each deoxynucleoside triphosphate, 1.0 μM of each primer, 1 U of TransStart FastPfu DNA Polymerase (TransGen, Beijing, China), and 4 ng genomic DNA. The PCR amplification program included initial denaturation at 94 °C for 3 minutes; followed by 23 cycles of 94 °C for 30 s, 60 °C for 40 s, and 72 °C for 60 s; and final extension at 72 °C for 10 minutes. PCR products were visualized in triplicate by electrophoresis on 1.0 % agarose gels and then compared to a DNA ladder to identify the bands of correct size. These bands were then excised and purified using the EZNA Gel Extraction Kit (Omega Bio-tek, Norcross, GA, USA) and quantified with the Qubit fluorometer. All replicates of each sample, in equal molar amounts, were pooled.

The sequencing library was prepared using the TruSeq DNA Kit (Illumina) according to manufacturer’s instructions. In accordance with the Illumina library preparation protocols, purified DNA was diluted, denatured, rediluted, and mixed with PhiX (equal to 30 % of the final DNA amount). The library was then sequenced with Reagent Kit v3 for 600 cycles in the MiSeq system, as described in the manufacturer’s manual. Using the Quantitative Insights into Microbial Ecology (QIIME) pipeline [11], the raw sequences were processed to concatenate reads into tags according to the overlapping relationship, then reads belonging to each sample were separated with barcodes and low quality reads were removed. The processed tags were clustered, and the operational taxonomic units (OTUs) were assigned to taxa by matching to the Greengenes database [12].

### FMT procedure

**Fig. 1:**
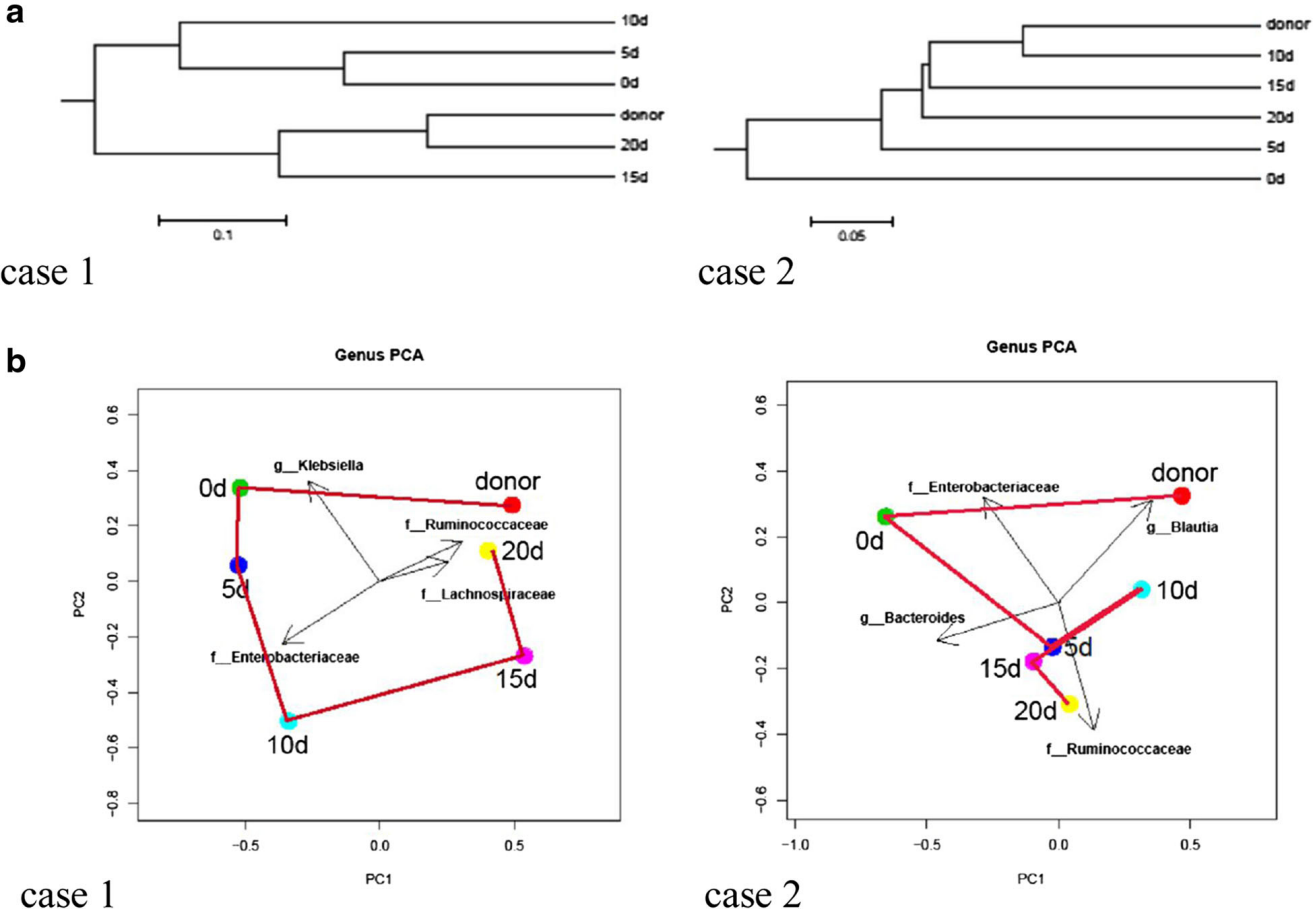
Changes in the gut microbiota of patients under fecal microbiota transplantation (FMT) treatment. **a** Clustering tree of weighted unifrac distances of microbial composition among different sampling time points. After receiving FMT, over time the gut microbiota of the two patients tended to approximate that of the donor. **b** Principal coordinate analysis (*PCA*) of the genus profile. The top four genera as the main contributors were determined and plotted by their loadings in these two components. *d* day

**Fig. 2:**
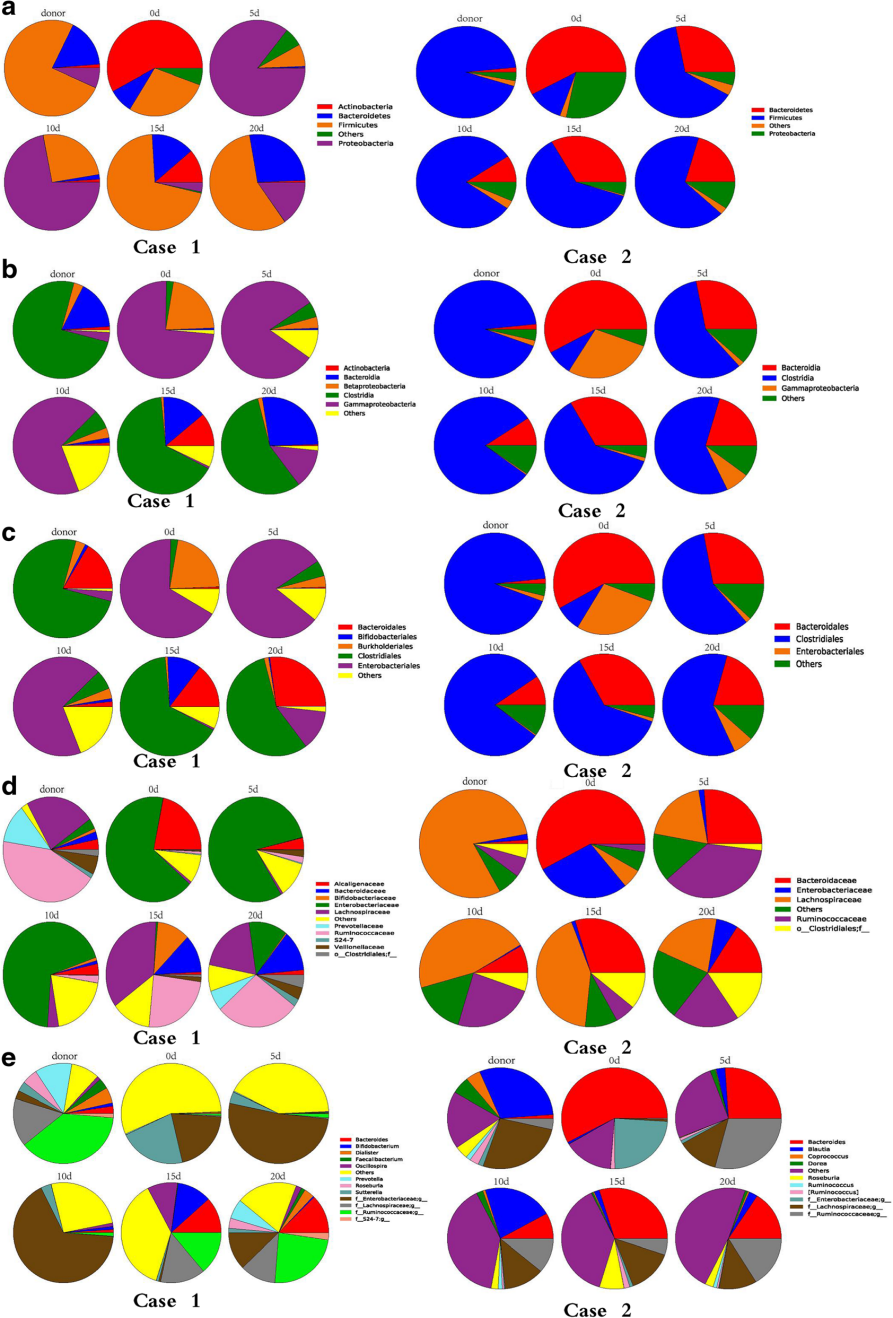
Predominant fecal microbiota composition in the donor and patients (case 1 and case 2) at the phyla level (**a**), the class level (**b**), the order level (**c**), the family level (**d**), and the genus level (**e**). Variations in the microbiota composition are shown at the representative time points in the days (*d*) following fecal microbiota transplantation

**Fig. 3:**
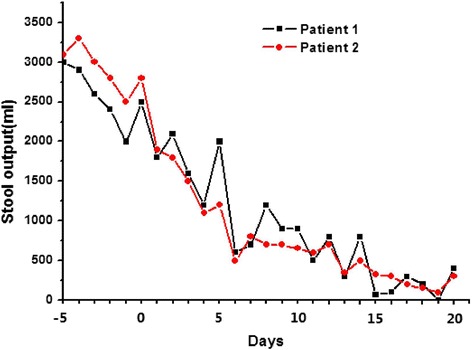
Volume of stool output in the two patients at time points (days) before and after fecal microbiota transplantation (*Day 0*)

**Fig. 4:**
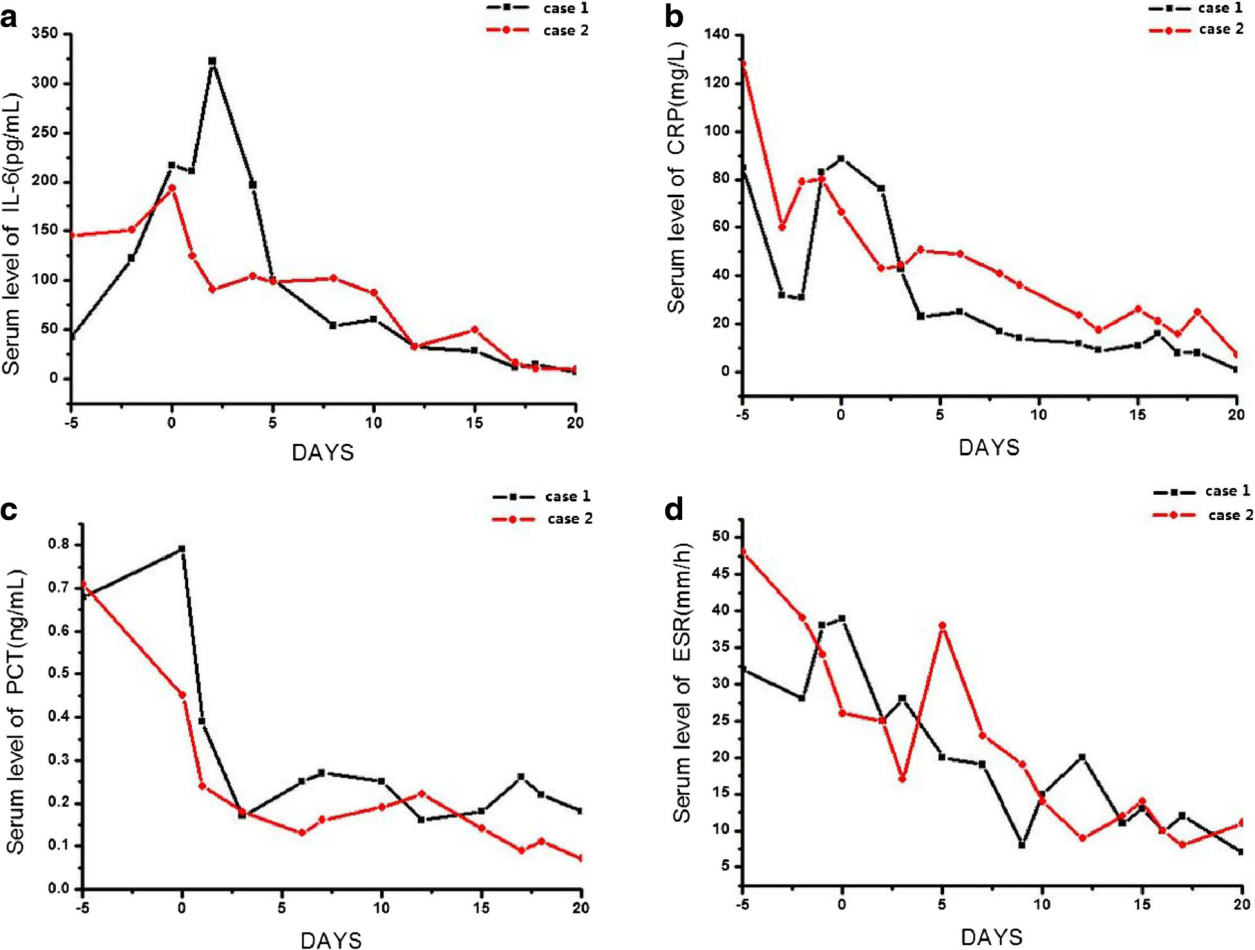
Serum levels of IL-6 (**a**), C-reactive protein (*CRP*) (**b**), procalcitonin (*PCT*) (**c**) and erythrocyte sedimentation rate (*ESR*) (**d**) in the two patients (case 1 and case 2) before and after fecal microbiota transplantation (*DAY 0*)

On analysis the fecal microbiota from the two patients were extensively perturbed (Fig. 2, day (d) 0) and therefore, we were very interested in trying to manage their microbiota disorders using FMT. The infusion of donor feces was performed in the two patients on day 0 (Figs. 1, 2, 3 and 4). The patients were each given FMT once. A graduate student (27 years old) was selected as the donor of fecal microbiota. He screened negative for blood-borne communicable diseases, and his fecal samples tested negative for common stool pathogens such as HAV-IgM, HBsAg, HCV-IgG, HDV-Ab, HEV-IgM, HIV, CMV, syphilis, roundworm, pinworm, hookworm, amoeba and duovirus, and so on. Fresh stool (150 g) collected on the day of infusion was diluted with 350 mL sterile saline. The homogenized solution was filtered twice through a pre-sterilized metal sieve. The filtrates (250 mL) were infused into each patient via a nasogastric tube. Each patient’s stool was collected every 5 days, and a 220-mg aliquot of each sample was immediately frozen at −80 °C until DNA extraction. We carried out 16 s RNA bacterial floral analysis of stool samples that were obtained by means of one stool sample every 5 days from each patient and the donor, and the methods and steps were the same as those described above.

### Serum inflammatory marker assays

Serum IL-6 was measured using enzyme-linked immunosorbent assay kits (R&D Systems, Abingdon, UK). The blood samples were collected and sent to the Department of Laboratory Testing in Daping Hospital for the detection of procalcitonin (PCT), C-reactive protein (CRP), and erythrocyte sedimentation rate (ESR). CRP (mg/L) was measured in capillary blood using the device QuikRead go CRP + Hb (Orion Diagnostica, Oy, Finland), which works on the principle of photometry and turbidimetry. ESR was measured using the Coulter GenS System (Coulter, Miami, FL, USA). PCT was measured using the immunochromatographic assay (Guangzhou Wondfo Biotech Co., Ltd. China).

### Statistical analysis

Correlation between two variables was tested by linear regression analysis with the Pearson test. The relative intensity of each band was calculated and expressed as a percentage of the sum of all fragments in the same lane of the gel. We also conducted OTU and principal component (PC) analyses with CANOCO software 4.5 for Windows (Microcomputer Power, Ithaca, NY, USA). A *P* value <0.05 was considered statistically significant.

## Results

### Changes in intestinal microflora following FMT

The proportion of nonpathogenic microbiota in the healthy donor feces (i.e., *Streptococcus*, *Bacillus coli*, *Streptococcus thermophilus*, *Bacillus acidi lactici*, *Lactobacillus plantarum*) was greater than that of the pathogenic bacteria (i.e., *Enterobacter cloacae*, *Yersinia enterocolitica*, *Klebsiella pneumoniae*). Consistent with the data obtained from the molecular phylogenetic tree, the OUT and PC analyses (Fig. 1a and b) both showed that the structure of the microbiome was dramatically changed in both patients compared to that of the donor. However, the gut microbiota structure in the patients showed a profound trend toward that of the donor feces, especially at 20 days post FMT in patient 1 and at 10 days post FMT in patient 2. The distance between the points representing the donor’s and patient’s microbiota decreased from 0.925 to 0.296 and from 0.637 to 0.289 in patient 1 and patient 2, respectively.

Compared to day 0, significant growth of bacteria in the Firmicutes phylum was noted at 20 days post FMT in both patients (Fig. 2a). In patient 1, the percentage of Actinobacteria was substantially decreased and became approximately equal to that of the healthy donor at 20 days post FMT. Bacteria in the Bacteroidetes and Firmicutes phyla increased and became approximately equal to that of the healthy donor at 15 days post FMT. There was an increasing trend for expression of Proteobacteria phylum genes by 5 days after FMT, and then it slowly decreased. Finally, it was probably stabilized at a level basically equivalent to that of the donor at 15 days after FMT. In patient 2, the populations of Bacteroidetes and Proteobacteria decreased to levels similar to those of a healthy donor by 10 days and 15 days post FMT, respectively (Fig. 2a).

Changes in the proportions of fecal microbiota at the class and order level were observed in both patients, and these altered proportions tended to follow those of the donor (Fig. 2b and c). The families Ruminococcaceae, Lachnospiraceae, and Veillonellaceae were significantly increased in patient 1 and contributed mostly to the boom of Firmicutes, while families Ruminococcaceae, unclassified Clostridiales, and Lachnospiraceae increased in patient 2 (Fig. 2d). The Proteobacteria families, Enterobacteriaceae and Alcaligenaceae, were significantly decreased in patient 1, while Enterobacteriaceae was significantly decreased in patient 2 (Fig. 2d). At the genus level (Fig. 2e), *Roseburia*, *Prevotella*, *Dialister*, *Faecalibacterium*, *Bacteroides*, and *Oscillospira* were increased in patient 1, with a rapid reduction of *Sutterella*. In patient 2, *Blautia* and *Roseburia* were increased after FMT, and *Bacteroides* was significantly decreased.

### Clinical outcomes

On the basis of the evidence of disturbed microbiota in the two patients, we applied FMT for the treatment of MODS and severe diarrhea, after conventional strategies with antibiotics and probiotics had failed. The patients’ septic symptoms and diarrhea were expected to improve after FMT. At day 1 post FMT, patient 1 had a decrease in body temperature from 39.6 °C prior to the infusion to 37.1 °C after infusion, and patient 2 had a decrease in body temperature from 38.3 °C to 37.3 °C. In the following days post FMT, neither patient had recurrence of MODS symptoms, and blood cultures from both patients remained sterile. Stool output from both patients declined after 7 days (Fig. 3), and their stools became well-formed. In patient 1, the fecal consistency became normal at 6 days post FMT. Stool frequency and volume returned to normal at 14 and 16 days post FMT in patient 1 and patient 2, respectively.

In addition, the serum levels of CRP, ESR, PCT and IL-6 in both patients were significantly decreased at day 20 post FMT, compared to those at day 0 (patient 1: CRP 88.3 vs. 1.0 mg/L, ESR 39 vs. 7 mm/h, PCT 0.79 vs. 0.18 μg/L, IL-6 216.9 vs. 6.7 pg/mL; patient 2: CRP 66.3 vs. 7.0 mg/L, ESR 26 vs. 11 mm/h, PCT 0.45 vs. 0.07 μg/L, IL-6 193.6 vs. 9.7 pg/mL). (Fig. 4). The level of consciousness in patient 1 improved, and he had normal respiration at 19 days post FMT. Patient 2 had a full recovery and was discharged from hospital.

## Discussion

The incidence of gastrointestinal infection, which results from MODS but also aggravates the disease, is relatively high in MODS patients, and gut microbiota imbalance may contribute to the infection [13]. Thus, FMT, which can help reconstruct the impaired gut microbiota barrier and correct the dysbiosis in patients in the ICU, has therapeutic potential for treating MODS [14, 15]. In this paper, we have reported two patients with sepsis, MODS, and severe diarrhea who were successfully treated by transplantation of fecal microbiota from a healthy donor. Our results are consistent with a previous case report of one patient with severe sepsis and diarrhea following vagotomy, who was successfully treated by FMT [16].

Recent studies have shown that the gut is the depot for bacteria and endotoxins associated with SIRS and MODS [7]. All elements of the gut - the epithelium, the immune system, and the microbiome - are impacted by critical illness, and can in turn propagate a pathologic host response [17]. Prior to FMT, the most notable changes in the fecal microbiota of patient 1 were increased Actinobacteria and decreased Firmicutes and Proteobacteria, and the most notable changes in the fecal microbiota of patient 2 were increased Bacteriodetes and Proteobacteria and decreased Firmicutes, compared with the fecal microbiota of the healthy donor. Because it makes up the largest part of the human gut microbiome and contains the genus *Lactobacillus* [18], the Firmicutes phylum could be beneficial to the normal function of the gastrointestinal tract. It is known that pathogenic bacteria translocate and proliferate more than the nonpathogenic ones, which can lead to gut barrier disruption, infection, and ultimately SIRS and MODS, by releasing multiple cytokines. The changes in microbiota composition observed in our patients could help further our understanding of the importance of maintaining a normal gut microbiota barrier and the feasibility of treating patients by restoring it.

Although FMT has been proposed as an effective method for correcting the dysbiosis among patients in the ICU and for restoring the normal gut microflora, its therapeutic role has not been completely explored [19, 20]. In our patients, the infection and diarrhea were alleviated by FMT, and the systemic immune response was also attenuated. The stool volume and frequency had increased significantly before FMT in both patients, but were gradually reduced after FMT. The stool was gradually formed. Here, we need to explain that at 3 days before FMT, we stopped all treatment with antibiotics in both of the patients so that the patients entered into an antibiotic washout period to prepare for later FMT. Because no antibiotics were present to further destroy the intestinal flora in this time range, the intestinal flora might recover somewhat. Therefore, the amounts of stool in the two patients fluctuated at 5 days before FMT. Sometimes the amounts were increased, sometimes they were decreased, and the overall trend was towards a decrease, but the stool frequencies in the two patients were still very high before FMT. In addition, there was no trend towards well-formed stool, and the overall composition of the stool was still similar to that of diarrhea. After being treated with FMT, the amounts of stool in the two patients were rapidly decreased, the stool frequencies were significantly decreased, and the stool gradually became well-formed. These findings indicate that the significant improvement in symptoms of diarrhea in the patients still depends on recovery of the balance of the intestinal flora due to FMT.

We observed decreased levels of IL-6, CRP, PCT, and ESR in both patients following FMT. An interesting phenomenon was seen in that although there was a trend towards a decrease in the overall levels of inflammatory factors such as IL-6 and CRP, the levels fluctuated somewhat in the first few days. A similar pattern was observed for PCT and ESR. However, all of these were minor changes. We hypothesize that these changes may be correlated with restoration of the intestinal flora and the improvement in clinical symptoms to some extent in patients after we stopped using antibiotics before FMT. The trend towards a decrease in these inflammatory factors also indicates that FMT might play an important role in the regulation of the immune system [21].

In an effort to identify a possible mechanism underlying the clinical benefits achieved in our patients, we evaluated the temporal changes in the microbiota composition following FMT. We further identified the variations in the composition of the microbiota in our patients following FMT under different OTU annotation levels. By comparing the fecal microbiota before and after FMT, we observed a transition to normal quantities (patient 1) or to a normal distribution (patient 2) of the aforementioned bacterial phyla found to be imbalanced (when compared with those in the healthy donor) before FMT, which is of valuable clinical significance. Changes in the proportions of fecal microbiota at the class, order, family, and genus levels were also observed in both patients following FMT, and these altered proportions tended to approach those of the donor. These findings suggest that reshaping the gut microbiota might be the fundamental mechanism of FMT in treating sepsis-associated MODS and that FMT is capable of restoring the normal quantities and/or the normal distribution of intestinal microbiota in patients with MODS.

## Conclusion

The main limitation of our study is that only two patients were included in our cohort, and thus it can serve only as a pilot investigation of the clinical application of FMT. We believe that our findings warrant future research with a larger cohort of patients to further explore the underlying mechanisms of the therapeutic effects of FMT and to define the appropriate conditions for its use. In conclusion, our results demonstrate the clinical effectiveness of FMT in treating patients with sepsis-associated MODS and diarrhea. FMT is capable of significantly alleviating septic complications by restoring the normal quantity and/or distribution of beneficial gut microbiota and correcting the dysbiosis among patients in the ICU. Thus, FMT shows promise as a putative novel therapy for MODS and diarrhea following severe sepsis.

## Abbreviations

CDI: *Clostridium difficile* infection
CRP: C-reactive protein
CT: Computed tomography
ESR: Erythrocyte sedimentation rate
FMT: Fecal microbiota transplantation
ICU: Intensive care unit
IL: Interleukin
MODS: Multiple organ dysfunction syndrome
OTU: Operational taxonomic units
PCA: Principal coordinate analysis
PCT: Procalcitonin
SIRS: Systemic inflammatory response syndrome
WBC: White blood cell
